# Regional Anesthesia Practices in Turkey During the COVID-19 Pandemic

**DOI:** 10.7759/cureus.10135

**Published:** 2020-08-30

**Authors:** Sevim Cesur, Can Aksu, Alparslan Kuş

**Affiliations:** 1 Anesthesiology and Reanimation, Kocaeli University, Kocaeli, TUR

**Keywords:** covid-19, regional anaesthesia, ultrasound-guided, peripheral nerve blocks, survey research

## Abstract

Introduction

The aim of the survey was to investigate the changes, methods, and preferences in regional anesthesia (RA) applications during the COVID-19 pandemic in Turkey.

Methods

The questionnaire prepared on surveymonkey.com was sent to anesthesiology and reanimation specialists by e-mail.

Results

A total of 126 physicians participated in the study. Forty-two point sixty-two percent (42.62%) of the participants reported an increase in RA practices in their clinical anesthesia applications, whereas 57.38% did not state any change. Neuraxial anesthesia was determined to be the most preferred RA application, with a rate of 74%. The distribution of peripheral nerve blocks (PNBs) showed that upper extremity blocks were used at a rate of 64.9%, lower extremity blocks at 30.38%, and trunk blocks at 15%. Investigation of neurostimulator (NS) and/or ultrasound (US) use with PNB showed that 44% of the participants used only US while 50% used both US and NS.

Conclusion

Neuraxial blocks play an important role in RA applications. PNB comprise one-quarter of RA applications during the pandemic. The importance of ultrasound has gradually increased in RA applications worldwide, as well as in Turkey, during the pandemic.

## Introduction

The outbreak of coronavirus-2 (SARS-CoV-2), which initially started in Wuhan, China, in December 2019, has turned into a global health problem. It has significantly impacted healthcare services and surgical volumes worldwide. The main clinical challenge results from the fact that approximately 80% of infected individuals either exhibit mild respiratory symptoms or are asymptomatic [1]. Due to the false negativity rate in clinical screening, it is difficult to reliably identify infected patients [1]. During the COVID-19 pandemic, patient management is based on patient safety and the protection of healthcare workers from infection. The virus can be transmitted even from asymptomatic carriers scheduled for surgery. One of the strategies to minimize the exposure to the virus is to avoid aerosol-generating procedures such as airway management, which is commonly utilized during surgery [2]. General anesthesia with airway intervention leads to aerosol generation, which exposes the healthcare teams to the risk of COVID‐19 infection during both tracheal intubation and extubation [3]. During tracheal intubation, the risk of the transmission of acute respiratory infections is thought to be 6.6 times higher among healthcare workers compared to those who are not exposed to it [4]. Regional anesthesia (RA), a non-aerosol-generating procedure, plays an important role during the pandemic when selected according to the patient and the type of surgery [5]. RA can provide adequate perioperative pain management by reducing opioid consumption as compared to general anesthesia. It reduces the risk of transmission of COVID-19 as a result of fewer postoperative pulmonary complications, nausea, and vomiting, reduced contact between the patient and healthcare worker, and early discharge [6].

In this study, we aimed to investigate the changes, methods, and preferences in regional anesthesia applications during the COVID-19 pandemic by reaching out to anesthesiology and reanimation specialists in Turkey by e-mail and through a survey study prepared on surveymonkey.com by the Turkish Society of Anesthesiology and Reanimation (TARD) and the Society of Regional Anesthesia (RAD).

## Materials and methods

Necessary permissions were obtained from the Kocaeli University Ethics Committee and Provincial Health Directorate for the study. The questionnaire prepared on surveymonkey.com was sent to anesthesiology and reanimation specialists who are members of TARD and/or RAD by e-mail. The purpose and voluntary basis of the survey were explained to all participants (Appendix 1). The survey, consisting of 17 questions, questioned the participants' demographics, the institution they received specialization training, the hospital they worked in, the years of professional experience, the rates of ultrasound (US) and/or neurostimulator (NS) use, regional anesthesia preferences, and the use of personal protective equipment depending on the type of surgery. The survey was conducted in Turkish. The collected data were kept confidential. Data were presented as numbers and percentages.

## Results

**Figure 1:**
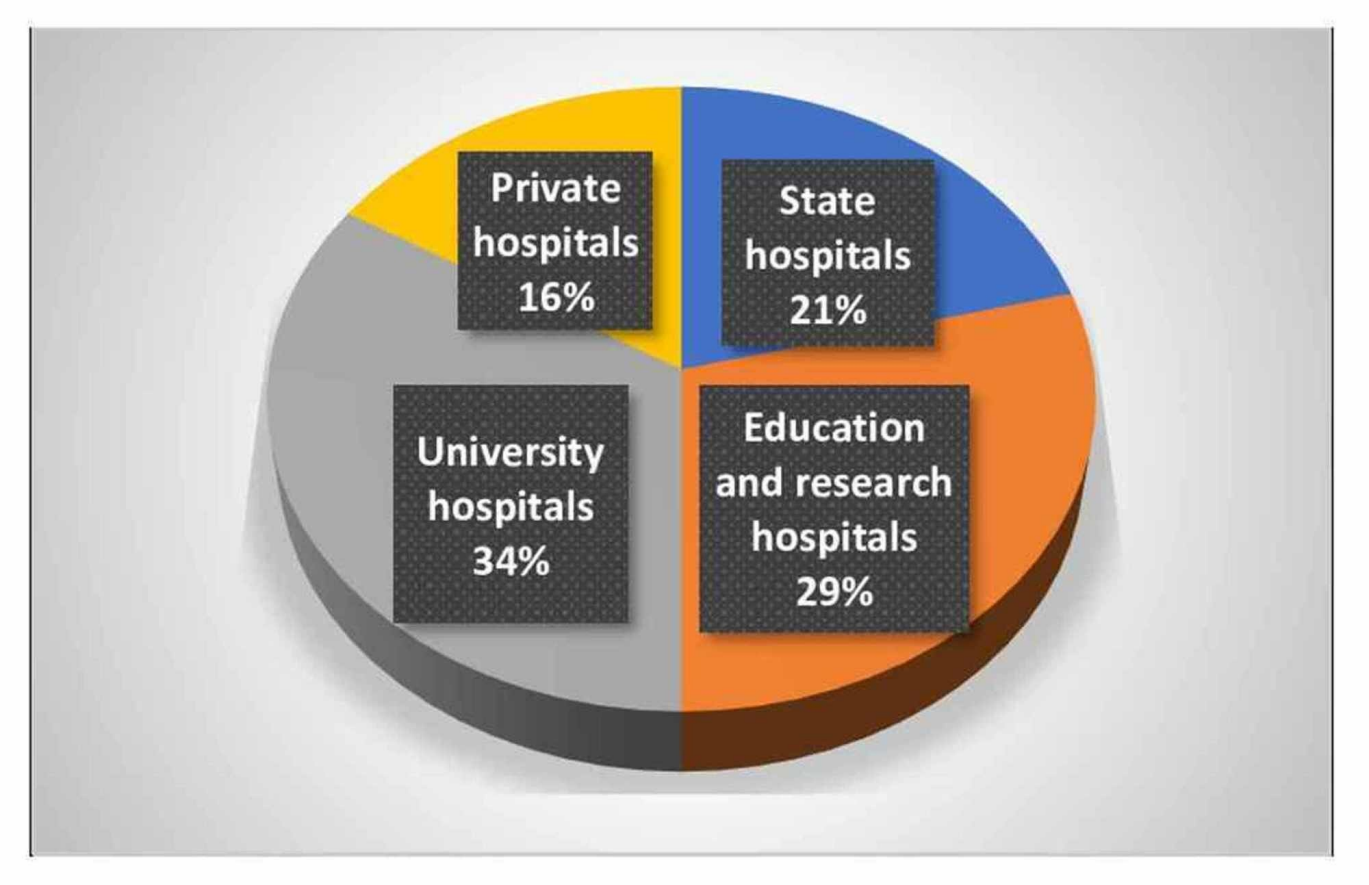
The hospitals where participants are currently working (%)

In May 2020, a total of 126 physicians who were sent the survey via surveymonkey.com and e-mail participated in the study. The participants' demographic data and the institutions they work in are given in Table 1 and Figure 1.

**Table 1:** Participants' demographic data Data are presented (min-max)

Number of participants surveyed	n: 126
Age (years)	44.1 (29-55)
Experience (years)	15.6 (1-24)

**Figure 2:**
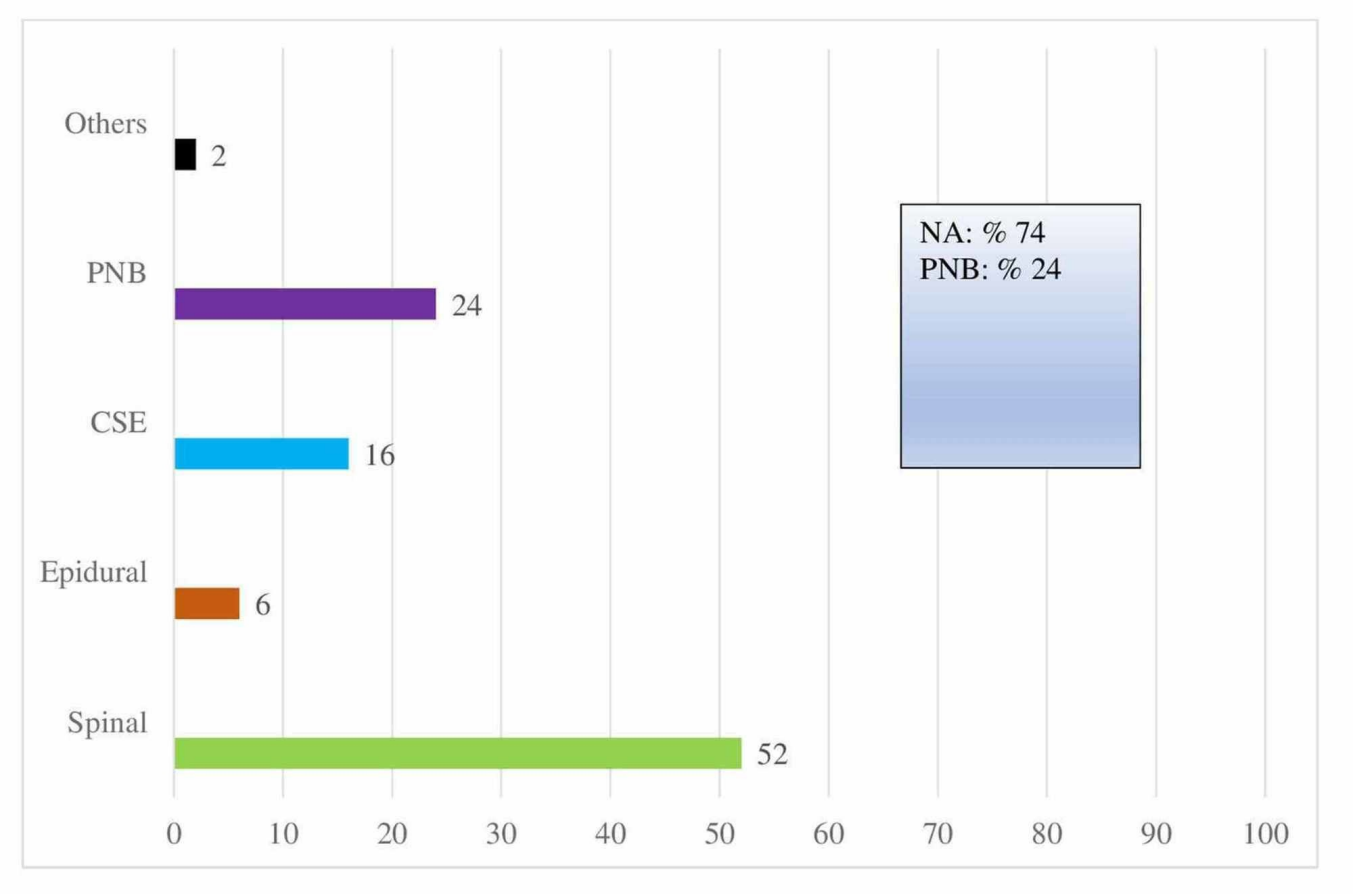
Distribution of regional anesthesia application in Turkey CSE: Combined spinal-epidural anesthesia, PNB: Peripheral nerve blocks, NA: Neuraxial anesthesia

Forty-two point sixty-two percent (42.62%) of the participants reported an increase in RA practices in their clinical anesthesia applications during the COVID-19 pandemic, whereas 57.38% did not state any change. Neuraxial anesthesia was determined to be the most preferred RA application with a rate of 74% (Figure 2). The distribution of peripheral nerve blocks (PNBs) showed that upper extremity blocks were used at a rate of 64.9%, lower extremity blocks at 30.38%, and trunk blocks at 15%.

The investigation of the neurostimulator (NS) and/or ultrasound (US) use with peripheral nerve blocks showed that 44% of the participants used only US, 50% used both US and NS, while the remaining 6% used only NS. Only 10% of the participants reported having a pressure monitor ready to apply PNB while 4% stated using a pressure monitor in their clinical practice.

**Figure 3:**
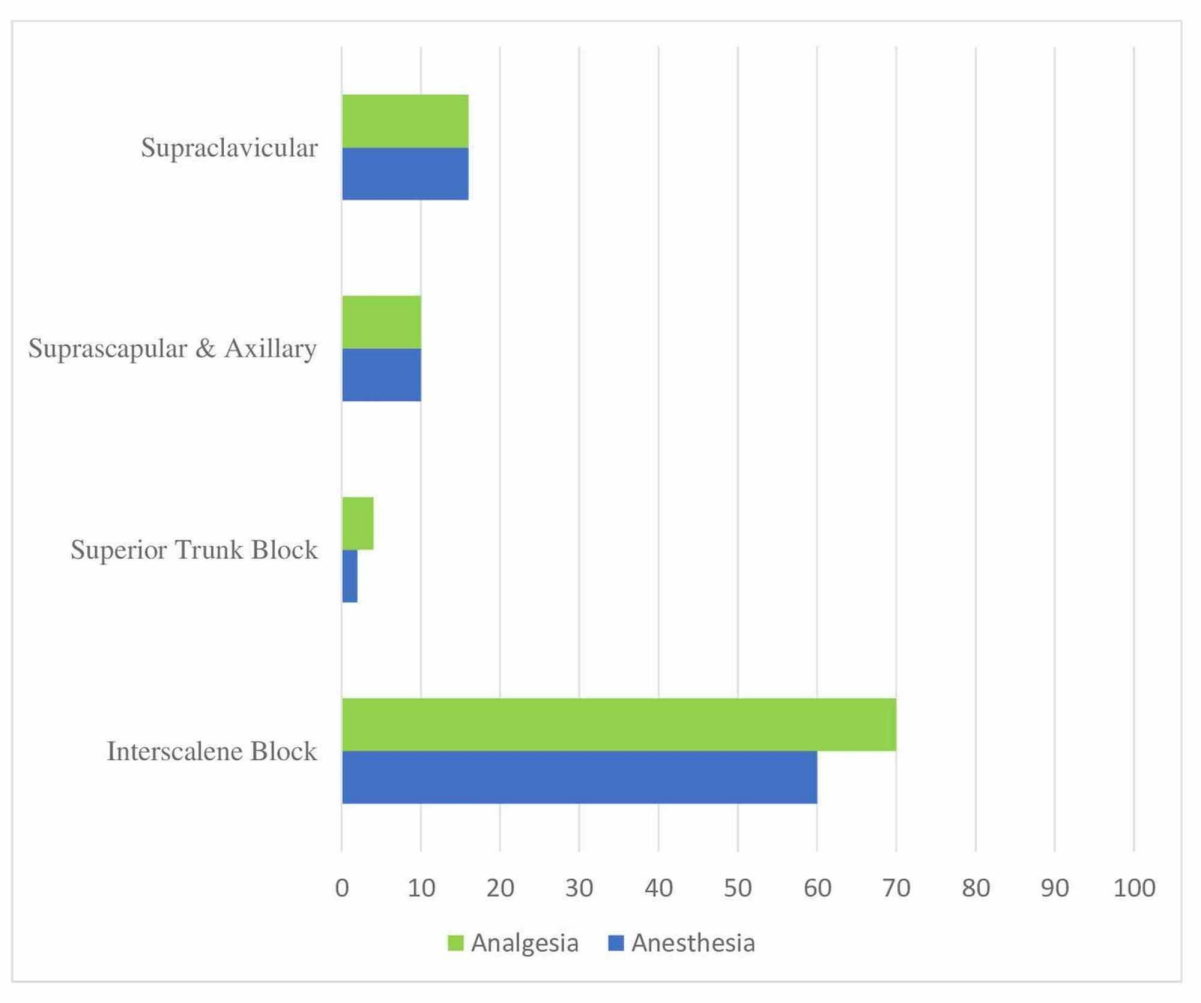
Distribution of applied RA techniques in shoulder surgery (%) Application rates of regional anesthesia techniques in confirmed/suspected COVID patients undergoing shoulder surgery RA: Regional anesthesia

When questioned about block preferences for analgesia and anesthesia in confirmed/suspected COVID patients undergoing shoulder surgery, 70% of the participants reported preferring the interscalene blocks for anesthesia/analgesia while 16% of the participants reported preferring the supraclavicular block (Figure 3).

**Figure 4:**
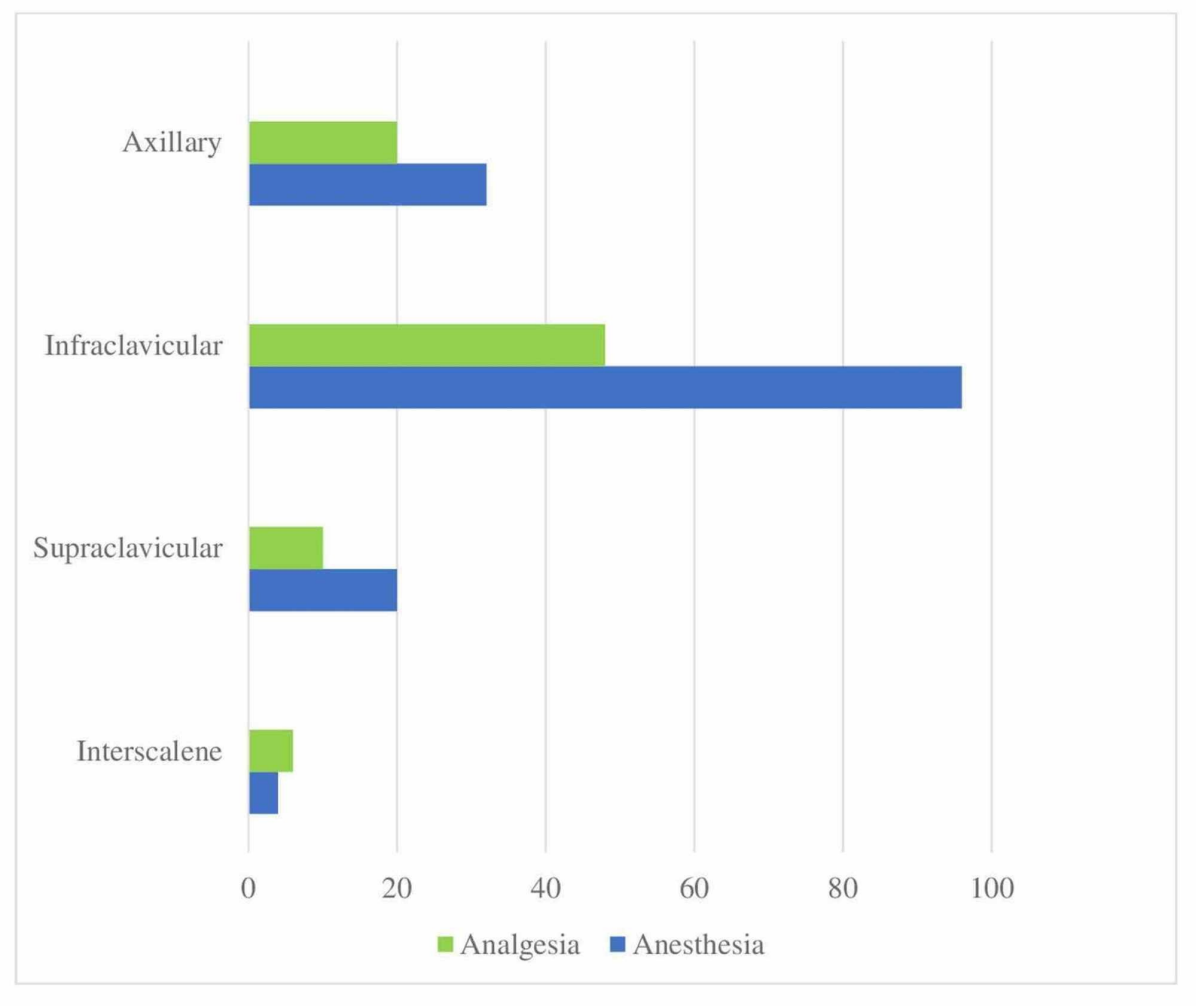
Distribution of applied RA techniques in hand surgery (%) Application rates of regional anesthesia techniques in confirmed/suspected COVID patients undergoing hand surgery RA: Regional anesthesia

When questioned about block preferences for analgesia and anesthesia in confirmed/suspected COVID patients undergoing hand surgery, the results showed a wide distribution, however, 96% of the participants reported preferring the infraclavicular block for providing anesthesia care (Figure 4). 

**Figure 5:**
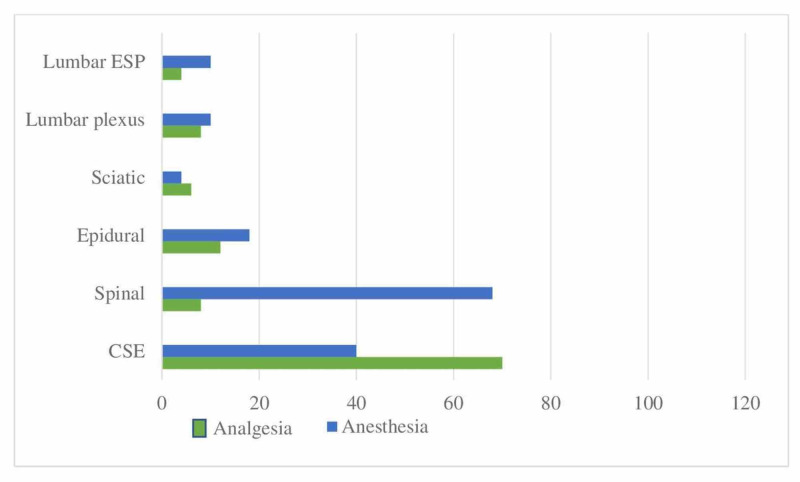
Distribution of applied RA techniques in hip surgery (%) Application rates of regional anesthesia techniques in confirmed/suspected COVID patients undergoing hip surgery ESP: Erector spinae plane block; CSE: Combined spinal-epidural; RA: Regional anesthesia

When questioned about block preferences for analgesia and anesthesia in confirmed/suspected COVID patients undergoing hip surgery, neuraxial methods were found to be in the foreground, with 68% of the participants preferring spinal anesthesia for providing anesthesia care (Figure 5).

**Figure 6:**
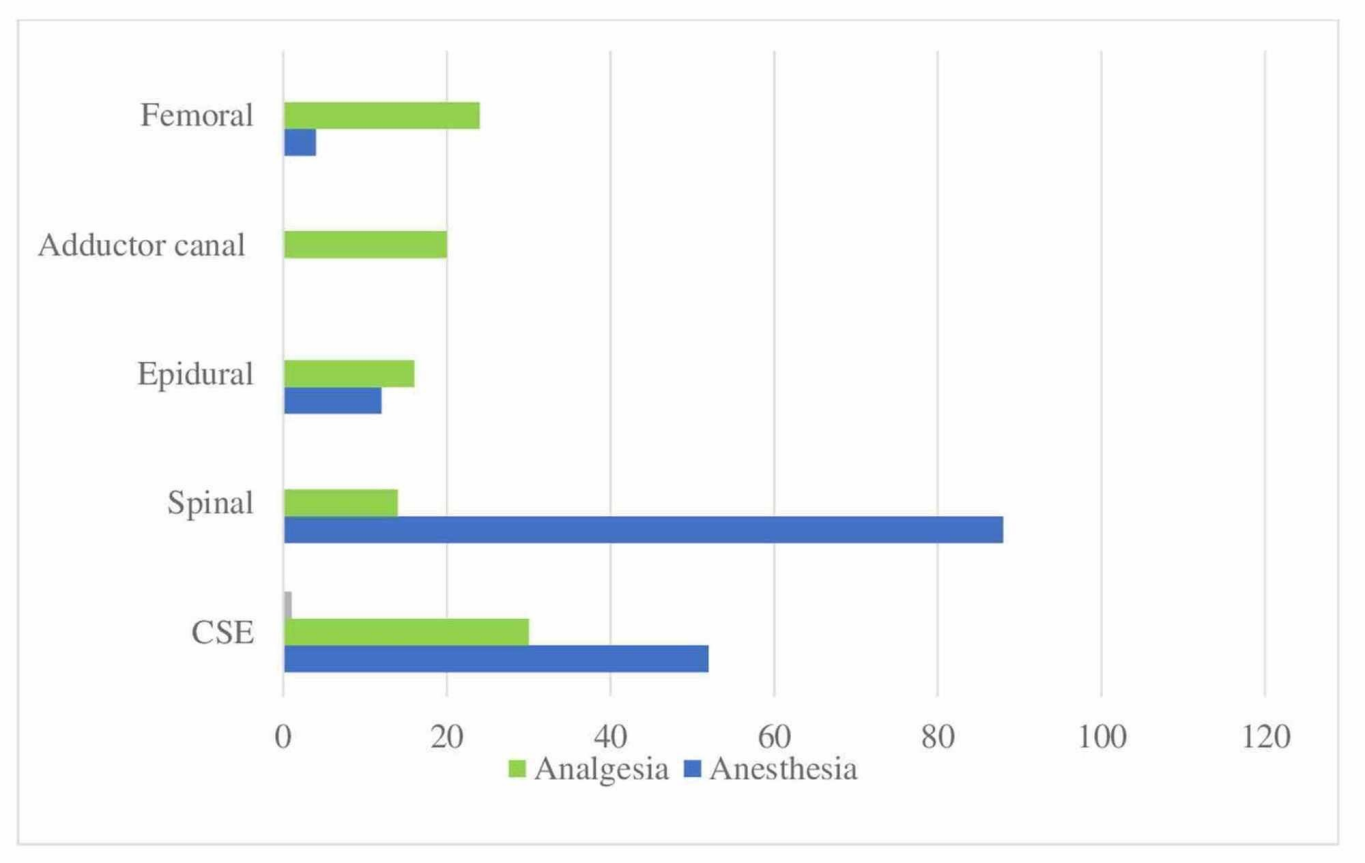
Distribution of applied RA techniques in knee surgery (%) Application rates of regional anesthesia techniques in confirmed/suspected COVID patients undergoing knee surgery. CSE: Combined spinal-epidural; RA: Regional anesthesia

When questioned about block preferences for analgesia and anesthesia in confirmed/suspected COVID patients undergoing knee surgery, neuraxial methods were found to be in the foreground, with 88% of the participants preferring spinal anesthesia for providing anesthesia care (Figure 6).

**Figure 7:**
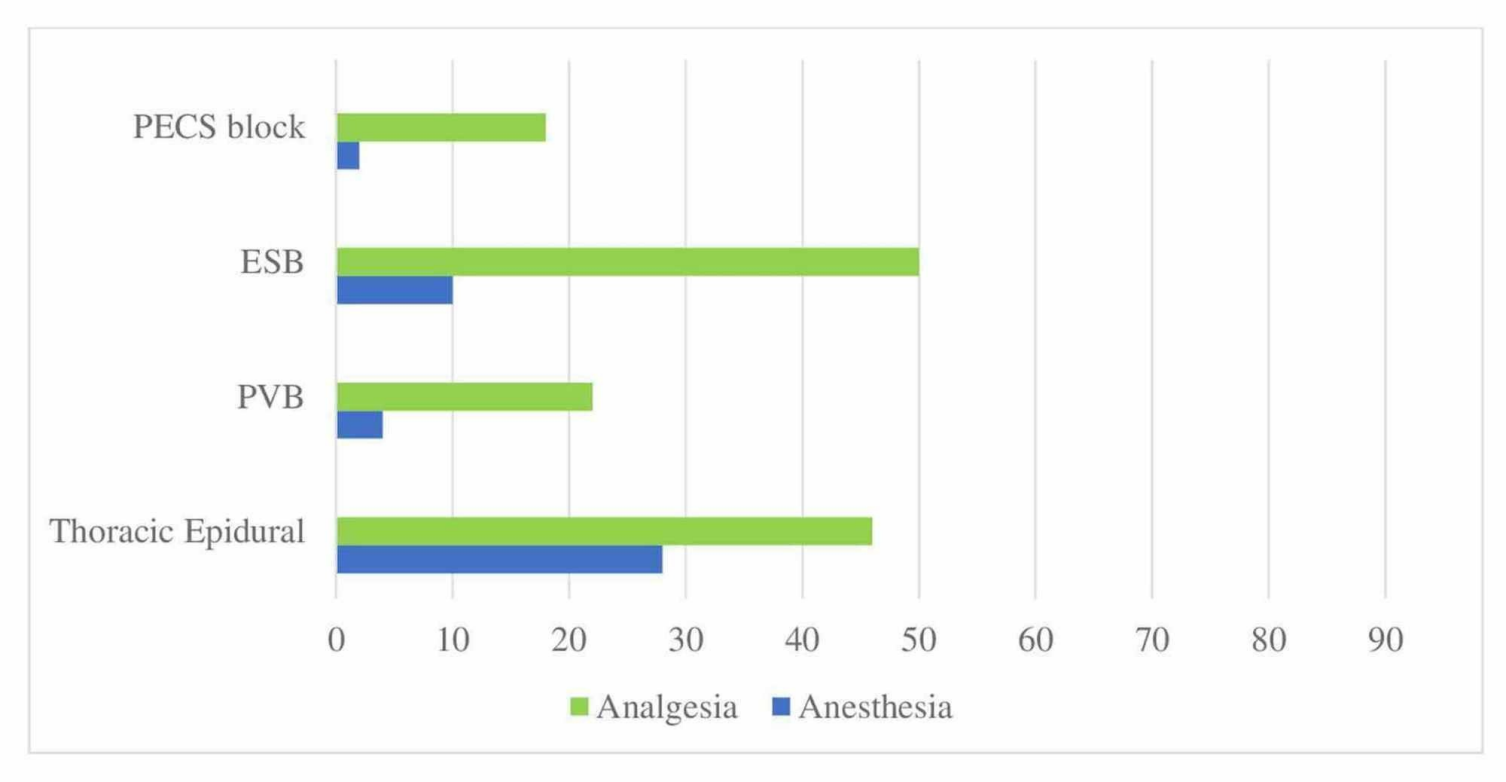
Distribution of applied RA techniques in thoracic surgery (%) Application rates of regional anesthesia techniques in confirmed/suspected COVID patients undergoing thoracic surgery PECS: Pectoral nerve blocks; ESB: Erector spinae plane block; PVB: Paravertebral block; RA: Regional anesthesia

The investigation of regional anesthesia methods preferred by the participants for patients scheduled for thoracic surgery revealed that the most commonly used method was erector spinae plane block (ESB) while thoracic epidural and paravertebral blocks were also used frequently for analgesia (Figure 7). 

**Figure 8:**
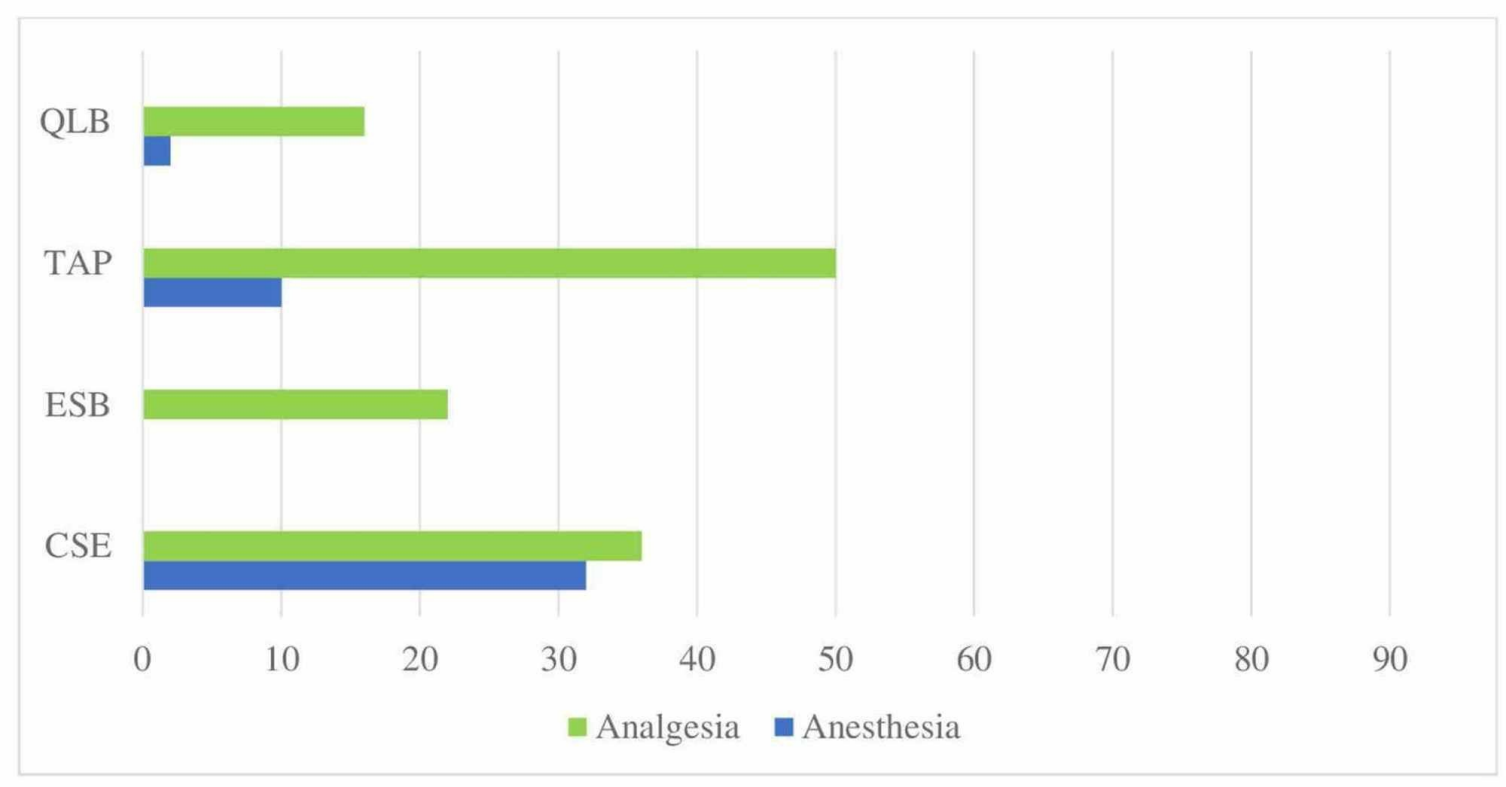
Distribution of applied RA techniques in abdominal surgery (%) Application rates of regional anesthesia techniques in confirmed/suspected COVID patients undergoing abdominal surgery QLB: Quadratus lumborum block; TAP: Transversus abdominis block; ESB: Erector spinae plane block; CSE: combined spinal-epidural; RA: Regional anesthesia

When questioned about block preferences for analgesia and anesthesia in confirmed/suspected COVID patients undergoing abdominal surgery, the transversus abdominis plane (TAP) block was found to be preferred most frequently for analgesia (Figure 8).

**Figure 9:**
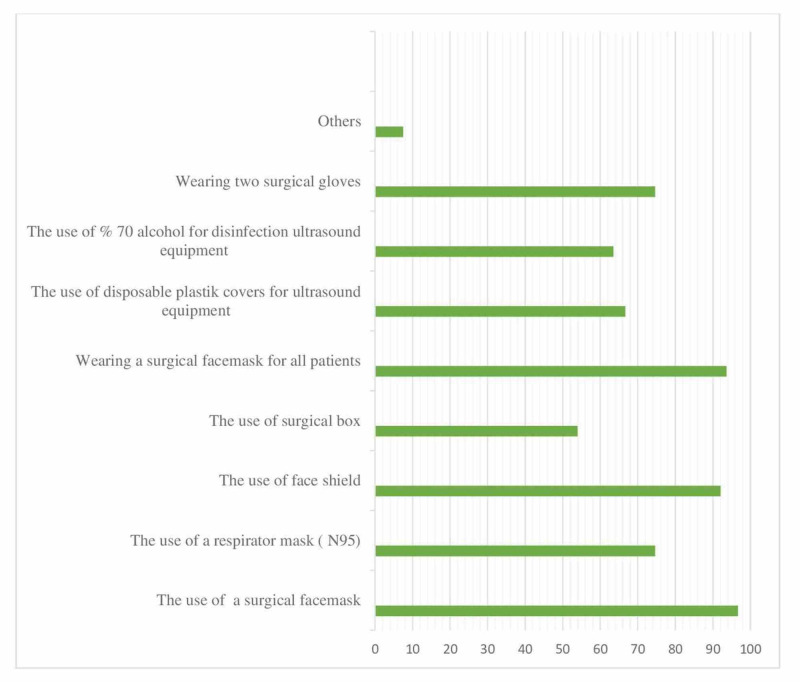
Distribution of personal protective equipment preferences for regional anesthesia (%)

When questioned about catheter preferences for postoperative analgesia, 45% of the participants reported not using a catheter while 55% reported using it, with an average removal of 1.9 days. The multiple-choice question questioning the precautions taken in RA practices during the pandemic demonstrated that 74% of the participants used N95 masks during the procedure while 93% stated placing surgical masks on patients. The measures taken are detailed in Figure 9.

## Discussion

General anesthesia with airway management, which results in aerosol-generating procedures, poses a risk to anesthetists during the COVID-19 pandemic. Various potentially aerosol-generating procedures that anesthesiologists may encounter include mask ventilation, tracheal intubation, extubation, non-invasive ventilation, disconnection of the ventilator circuit during use, use of open tracheal aspiration system, etc. Considering all these risks, on the other hand, RA lowers the risk of postoperative complications, and this becomes more important in the context of ongoing respiratory infection [7-8]. Regional anesthesia may be the preferred choice for providing anesthesia care when possible, as it can provide an alternative safe anesthetic care plan by avoiding the need for aerosol‐generating procedures [2].

Forty-two point six (42.6%) of the participating physicians reported an increase in RA use during the pandemic. This increase may be associated with the advantages of RA techniques as well as the fact that the physicians participating in the survey are among those who are responsible for RA. Recently, the integration of ultrasound with RA has increased the success rate of blockades, thus resulting in a higher interest in RA. It was determined that the physicians who participated in the survey study used ultrasound at a rate of 94.8% while using NS and US dually at a rate of 50%. In Turkey, a study conducted by Gürkan et al. in 2014 reported the use of ultrasound at a rate of 50%, with a higher incidence in training, research, and university hospitals [9]. A study conducted in Canada in 2011, in which ultrasound was found to be used more frequently in academic educational institutions (39%), reported similar results to those of ours [10]. In 2015, a study conducted in Greece reported NS use with peripheral nerve blockade at a rate of 78.5% [11]. Our study results show that US use has gained significant popularity at this point. Since there is a homogeneous distribution among the institutions where the respondents work, we can say that US is now widely used not only in academic educational institutions but also in public and private hospitals.

The current study aims to provide further insight into the clinical use of RA by anesthesiologists. The answers given by the participating physicians demonstrated that neuraxial blocks (74%) were used most frequently for providing RA, followed by peripheral nerve blocks (24%). A domestic study reported the use of peripheral nerve blocks as 12% in 2014 while a Greek study reported a rate of 11.66% in 2015, which supports the idea that peripheral blockade has grown in popularity due to the increasing prevalence of US use, along with enhanced knowledge and interest in RA [9,11].

Among other neuraxial techniques, spinal anesthesia was the most commonly used RA application due to its motor blockade and rapid onset. The examination of the peripheral block distribution reveals that upper extremity blocks are preferred most frequently, followed by lower extremity and trunk blocks, respectively. The fact that lower extremity blocks are preferred less frequently than upper extremity blocks may be due to technical difficulties in applying deep blocks, lower success rates, and the requirement of at least two plexus blocks in major surgery [12]. Additionally, spinal and epidural anesthesia, which are proven to be effective and frequently performed by anesthesiologists, are important competitors as an alternative to lower extremity peripheral nerve blocks.

When questioned about block preferences according to the type of surgery, it was found that 70% of the participants used interscalene blocks for anesthesia/analgesia in shoulder surgery. A superior trunk block (4%) was found among the least preferred blocks in shoulder surgery. A superior trunk block may come to the fore since phrenic nerve palsy should be avoided as much as possible in patients with confirmed/suspected COVID [13]. The incidence of hemidiaphragmatic paresis after an interscalene brachial plexus block can be as high as 100% with local anesthetic volumes of 20 ml or more [14-15]. This incidence can be reduced by up to 45% by reducing the local anesthetic volume to 5 ml or 10 ml [14,16]. In their study, Kim et al. reported the incidence of phrenic nerve palsy using superior trunk blocks with 10 ml of local anesthetic to be as low as 4.8% [17]. Additionally, an ultrasound-guided interscalene brachial plexus block is usually performed at the level of the cricoid cartilage where the C5 and C6 roots are located in the groove between the anterior and mid-scalene muscles. The enveloping fascial layer around the hypoechoic C5 and C6 roots is very thin, which increases the risk of a subepineurial injection and injury from needle-nerve contact. Unlike the C5 and C6 roots, the superior trunk is surrounded by a clearly visible and well-defined connective sheath. This not only facilitates target visualization and identification but also enhances resilience to needle-nerve contact [17].

Although it is considered to be more advantageous in terms of being a more superficial block, with a better-defined connective tissue sheath and less incidence of phrenic nerve palsy, we believe that it would be more rational if anesthesiologists used the RA techniques they have the most experience in during the pandemic.

When the participants' preferences in hand surgery were questioned, the infraclavicular block was found to rank first with a rate of 96%, followed by the axillary block. As suggested by the COVID-19 regional anesthesia guidelines of the American Society of Regional Anesthesia and Pain Medicine (ASRA) and the European Society of Regional Anaesthesia & Pain Therapy (ESRA), we see that participants prefer an infraclavicular and axillary block that least affects respiratory functions [18].

When questioned about their preferences in thoracic and abdominal surgery, the participants were found to use the ESB and TAP blocks most frequently. If the block is to be performed under general anesthesia during the pandemic, it is recommended to decide on block preferences so as not to require repositioning since the tracheal tube may be disconnected and risk exposure to aerosol [19]. Considering these risks, the participants' preferences for ESB in thoracic surgery and a TAP block in abdominal surgery are in line with the general recommendations.

When questioned about their catheter preferences for postoperative analgesia, it was found that 45% of the participants did not prefer using a catheter while 55% did. Although a patient control analgesia (PCA) pump requires healthcare professionals to frequently contact with patients, pain management with opioid-free analgesia can be advantageous in patients with respiratory comorbidities. Therefore, catheterization should be determined according to the patient and the type of surgery.

Seventy-four percent (74%) of the respondents reported wearing N-95 masks during RA practices and 93% stated that they place surgical masks on patients. While it is recommended to wear surgical masks while applying regional anesthesia during the COVID-19 pandemic, our results also exhibit a quite high rate (96%) in this regard. Physicians are advised to use N-95 masks during the block procedure applied in close contact with the patient [19]. It was observed that 74.6% of the participants preferred using N-95 masks while 92% used face visors. Sixty-five percent (65%) of the participants made sure the US device and its probe were covered with sheaths.

A total of 126 physicians participated in the survey. This number may not be sufficient enough to reflect the whole country. The physicians filling out this questionnaire may also be those who have the most interest in and are responsible for RA practices. Some of the participating physicians may also be colleagues working in the same clinic who received training from similar educational institutions. Although the institutions where the participants work exhibit a homogenous distribution, the results may not accurately reflect RA applications in Turkey; however, we believe that these data can be useful in terms of providing an insight into the applications used during the pandemic.

## Conclusions

In conclusion, neuraxial blocks play an important role in regional anesthesia applications. Peripheral nerve blocks comprise one-quarter of RA applications during the COVID-19 pandemic. The importance of ultrasound has gradually increased in RA applications worldwide as well as in Turkey during the pandemic. The pandemic has also made some contributions to our clinical practice. It is important to proceed with national training programs and establish guidelines, particularly on regional anesthesia. Official healthcare providers should take the necessary precautions in international outbreaks, such as pandemics, and enforce and follow measures regarding personal protective equipment.

## Appendices

Appendix 1

Regional Anesthesia Practices in COVID-19 Pandemic Survey

1. Age

2. Year of experience

3. Working hospital

State Hospital

University Hospital

Other (Please Specify)

4. Which of the following devices do you have in your clinic?

Ultrasound

Neurostimulator

Pressure Monitor

5. Please specify the General Anesthesia and Regional Anesthesia application ratios since the Pandemic was announced.

Regional Anesthesia ...%

General Anesthesia ...%

6. Has there been a proportional increase in the use of RA in your routine applications?

Yes

No

7. Please indicate the percentages of your RA applications according to purpose

Surgical Anesthesia ...%

Postoperative Analgesia ...%

Other (Please specify) ...%

8. What are the approximate percentage distributions of your RA applications during this pandemic?

Spinal Anesthesia ...%

Epidural Anesthesia ...%

CSE ...%

Peripheral Nerve Blocks ...%

Other (Please specify) ...%

9. Please indicate which blocks you use in your peripheral nerve block applications in which percentages.

Upper Extremity ...%

Lower Extremity ...%

Truncal Blocks ...%

Paraspinal Blocks ...%

10. Which block do you apply/prefer for a patient who is COVID (+)/suspected and will undergo knee surgery? For what purpose?

Anesthesia

Analgesia

CSE

Spinal Anesthesia

Adductor Canal Block

Femoral Block

Other (Please specify)

11. Which block do you apply/prefer for a patient who is COVID (+)/suspected and will undergo hip surgery? For what purpose?

Anesthesia

Analgesia

CSE

Spinal Anesthesia

Epidural

Sciatic Block

Lumbar Plexus Block

Lumbar ESP

PENG Block

Other (Please Specify)

12. Which block do you apply/prefer for a patient who is COVID (+)/suspected and will undergo shoulder surgery? For what purpose?

Anesthesia

Analgesia

Interscalene Block

Superior Trunk Block

Suprascapular & Axillary Block

Supraclavicular Block

Other (Please specify)

13. Which block do you apply/prefer for a patient who is COVID (+)/suspected and will undergo hand surgery? For what purpose?

Anesthesia

Analgesia

Interscalene Block

Supraclavicular Block

Infraclavicular Block

Axillary Block

Other (Please specify)

14. Which block do you apply/prefer for a patient who is COVID (+)/suspected and will undergo thoracic surgery? For what purpose?

Anesthesia

Analgesia

Thoracic Epidural

Paravertebral Block

Erector Spinae Plane Block

Pectoral Blocks

Other (Please specify)

15. Which block do you apply/prefer for a patient who is COVID (+)/suspected and will undergo abdominal surgery? For what purpose?

Anesthesia

Analgesia

CSE

TAP block

QLB

ESP Block

16. Do you use catheters during the pandemic? If yes, how many days do you use the catheter?

Yes

No

17. Do you take additional precautions during RA applications; if yes, what are these?

N95 (FFP3) masks

Eye protection

Surgical gown

Double glove

Surgical mask to patients

Using sterile paper drapes for USG

Using plastic covers for USG

Other (Please specify)
